# The Effect of Cumulative Dose of Glucocorticoids on Bone in Children with Inflammatory Bowel Disease (3-year Follow- up study)

**DOI:** 10.1186/1687-9856-2013-S1-P168

**Published:** 2013-10-03

**Authors:** S Tomková, E Majorová, P Vanuga, J Payer

**Affiliations:** 1 Osteocentrum, Hospital Šaca, Košice, Slovakia; 2 Children´s Faculty Hospital, 2nd Department of Pediatrics, Košice, Slovakia; 3 V. Dept of Internal Medicine, Fac Hospital, Bratislava, Slovakia; 4 Dept of Endocrinology, National Institute of Endocrinology and Diabetology, Lubochna, Slovakia

## Background

The aim of the study was to evaluate the effect of cumulative dose of glucocorticoids during 3 years on bone mineral density (BMD) and bone turnover (BMT) measurement in children suffering from inflammatory bowel disease (IBD).

## Patients and Methods

In cohort of 64 children with IBD (54 children with Crohn disease and 10 with Ulcerative colitis) we measured bone mineral density at the time of diagnosis and two times during follow-up. Bone turnover /osteocalcin (OC) and C-terminal telopeptide of type I collagen (CTx)/ was measured using ELISA same time as BMD. All patient were treated by glucocorticoids with average of cumulative dose 4,46g. The obtained results were analyzed using correlation analysis.

## Results

At the time of diagnosis we observed the total body BMD Z-score –1,7 (mean **±** SD). There was no change during the treatment (1,77 resp. 1,69 Z-score). The level of CTX and osteocalcin paradoxically increased, but there was no statistical significance.

## Conclusion

In our study we didn’t find correlation between BMD, cumulative dose of glucocorticoids, CTX and osteocalcin during period of 3 years. Insignificant increase of bone markers was established. According to our results we can speculate that bone status impairment of bone mass is independent in children with IBD on treatment with glucocorticoids regarding to inflammation itself.

